# Electromyographic Evaluation of Grip Force and Anthropometrics of the Dominant Hand in Cricketers

**DOI:** 10.7759/cureus.104877

**Published:** 2026-03-08

**Authors:** Manish Rathod, Ruchi Kothari, Shreyash Yedke, Pradeep Bokariya

**Affiliations:** 1 Physiology, Mahatma Gandhi Institute of Medical Sciences, Wardha, IND; 2 Anatomy, Mahatma Gandhi Institute of Medical Sciences, Wardha, IND

**Keywords:** anthropometrics, cricketers, electromyography, grip force transducer, hand grip

## Abstract

Background

Cricket is a physically challenging sport with multiple specializations that call for different levels of fitness. Cricket stands as the second most popular sport being played worldwide. In the game of cricket, during gripping activities, flexor muscles of the hand create grip force, and the assessment of grip force is useful to explore the batting posture diversity of batsmen. Grip force can be assessed by handgrip strength (HGS), which serves as a vital measure of the muscular power of the hand. Arm anthropometrics is an array of indices to measure muscle and fat content by measuring the arm dimensions. Electromyography (EMG) is used to assess the electrical activity of the flexors of the hand during voluntary contraction and gives an idea about the grip of a person. Linking both EMG and anthropometrics with the hand grip function test of the dominant hand of a cricketer could provide a comprehensive, all-around fitness assessment at one go.

Methodology

The study design was descriptive and cross-sectional, carried out as a collaborative project performed in the anatomy and physiology departments of a rural medical college. A total of 80 participants (40 cricketers and 40 age-matched controls) within the age group of 17-25 years were included in the study using the convenience sampling technique. The study participants were evaluated for EMG and arm anthropometrics of the dominant hand. Unpaired Student's t-test and Pearson's correlation test were utilized for the data analysis and to determine the relationship among the study variables.

Results

The mean age of cricketers was 20.08 ±0.81 years, and controls were 20.00 ± 0.69 years. The difference between means of age, height, weight, BMI, systolic blood pressure (BP), and diastolic BP was not statistically significant. Cricketers had significantly higher values of arm anthropometric parameters, such as mid-upper arm circumference (MUAC), mid-upper arm area (MUAA), mid-upper arm muscle circumference (MUAMC), mid-upper arm muscle area (MUAMA), corrected mid-upper arm muscle area (CMUAMA), and body fat % than those of controls. On correlation analysis of mean HGS, a statistically significant positive association with MUAMA (r value = 0.42, p = 0.005) was found, with statistically significant negative correlation with TSF (r = -0.39, p = 0.01) and with an arm fat index (AFI; r = -0.40, p = 0.008). On correlation analysis of the root mean square (RMS) of EMG, there was significant correlation with all arm anthropometrics, such as MUAC (r = 0.47), MUAA (r = 0.48), MUAMA (r = 0.41), mean HGS ( r = 0.39), and max HGS (r = 0.47).

Conclusion

A cricketer's grip force assessment has a crucial role in preventing injuries and maintaining overall fitness. The present study demonstrated that arm anthropometrics, HGS, and RMS of EMG are significantly greater in cricket players in comparison to controls. The latter two variables showed good correlation with the majority of the arm anthropometric variables recorded. The data of mean HGS, max HGS, mean RMS EMG, and various arm anthropometrics derived from this research could serve as a reference for comparison of healthy and injured cricketers. Training programs that emphasize HGS and anthropometric evaluation at different levels, such as institute, university, state, and national, may be planned in order to improve the overall performance of cricketers.

## Introduction

Cricket is an established team sport that has been around for ages and is very popular, particularly in the Indian subcontinent. A highly demanding form of the game in the current era requires a cricketer to possess a superior hand grip and stronger arms to make an exceptional performance. In Twenty20 (T20)-like one-day events, batsmen need training with credible emphasis on the development of upper extremity power, which is especially required for powerful cricket shots and for seamless bowling.

It is a game that requires excellent upper extremity power and coordinated movements of the shoulder, arms, and wrists [1]. An optimal grip of the batsman is especially significant while displaying outstanding cricket knocks. The forearm strength is quintessential for not only batting but also for throwing the ball while fielding and bowling [2]. Different bowling actions access different muscular movements of the upper arm. For instance, the swing of the ball performed by seamers and the spin performed by spinners involve wrist rotation by the flexor carpi radialis and ulnaris muscles, along with the palmaris longus. While performing any gripping actions, the flexor muscles of the hand are responsible for the grip, while the extensors of the forearm serve to stabilize the wrist [1,3].

For cricketers, the structures most frequently injured are the rotator cuff and glenoid labrum of the upper arm, as they are subjected to repetitive and forceful throwing actions involved in fielding and bowling in cricket. Therefore, to maintain a cricket player's shoulder region free from pain, a considerable amount of dynamic force of the upper arm is necessary, which, to a large extent, lies on the stabilizing muscles [4].

Although shoulder injuries, such as rotator cuff and glenoid labral pathology, are common in cricketers due to repetitive overhead actions, upper limb function operates through a kinetic chain mechanism. Cricket actions, such as bowling, throwing, and batting, involve a coordinated kinetic chain, where force is generated from the lower limbs and trunk, transmitted through the shoulder complex, and ultimately expressed distally through the forearm and hand. Efficient energy transfer across this chain depends on both proximal stability and distal force production. The shoulder functions as a dynamic stabilizer during high-velocity upper limb actions by contributing to optimal force transfer and neuromuscular coordination of the shoulder complex. Therefore, assessment of distal muscle function using handgrip strength (HGS) and electromyography (EMG) may provide indirect insight into the overall upper extremity performance and injury risk in cricketers.

EMG is used to investigate skeletal muscle's electrical activity that can be assessed with surface electrodes. EMG is utilized for the quantification of muscle activation during isometric (static) and isotonic (dynamic) contractions. Owing to its non-invasive nature, it can be employed in the field of research, having various clinical applications. Surface EMG is the recording of the action potentials elicited by functional motor units located on the surface of the skin. For the evaluation of the performance of upper limb muscles, surface EMG (sEMG) has been known to be a valuable tool [5]. Kinesiological EMG involves the estimation of muscular action with limb movements, so this type of evaluation is best suited for cricketers. EMG of voluntary contraction of the flexors of the hand is crucial to have an idea about the grip of a person.

The electrical activity of elbow flexors and extensors has been previously investigated among cricket players [6-8], with recent work focused on bowlers, where muscle action while bowling has been studied. Physiological assessment using EMG for the analysis of musculature is important for cricketers, but its correlation with HGS is deficient in the literature.

To generate grip force, the hand and forearm muscles play an integral role. Grip force is defined as the force generated during powerful flexion of all finger joints, representing the optimum voluntary force an individual can produce with the hands under normal biokinetic conditions [9]. The hand is very prone to musculoskeletal disabilities, resulting in upper limb injuries because of its dexterity utilized in different sports [10].

A cricketer's grip force assessment has a crucial role in preventing injuries and maintaining overall fitness, but it is often not addressed. Measurement of HGS is paramount in evaluating the efficacy of hand care strategies and rehabilitation.

Arm anthropometrics is an array of indices assessing the shape of the arm used as a proxy measure of muscle and fat content by measuring the arm dimensions. Two major parameters included in this term are mid-upper arm circumference (MUAC) and triceps skinfold (TSF) thickness, which are further employed to derive other indices [11-13].

Some previous studies [14,15] have ascertained its link to arm anthropometric indices such as MUAC, mid-upper arm area (MUAA), mid-upper arm muscle area (MUAMA), etc. across athletic populations. Physical health requirements can be modified to enhance the productivity of cricketers by trainers who would periodically assess their arm anthropometrics. Training programs that emphasize HGS and anthropometric evaluation at different levels, such as institute, university, state, and national, may be planned in order to improve the overall performance of cricketers.

As for the best available estimate, there is a lack of evidence on the relationship between EMG of the flexor muscles of the hand and different arm anthropometric indices.

Hence, the current research was conceived with the intent to explore the unleashed interrelation between the study parameters. The primary objective of this study was to compare the dominant HGS, sEMG of forearm flexors, and arm anthropometric indices between young male cricketers and age-matched non-athletic controls. The secondary objectives were to quantify the correlation between muscle mass indices and the HGS and to investigate the relationship between electrical muscle activity (RMS of EMG) and physical anthropometric dimensions.

## Materials and methods

Study design and setting

The study design was descriptive and cross-sectional. It was collaborative research performed in the anatomy and physiology departments of a rural medical institute located in central India.

Sample size and sampling technique

A total of 80 participants (40 cricketers and 40 age-matched controls) within the age range of 17-25 years were included using a convenience sampling technique. The sample size for comparing the means of two independent groups was calculated by assuming a moderate effect size (d) of 0.6, α (type I error) of 0.05, and power (1−β) of 80%. Inserting these values in the OpenEpi 3.01 statistical software (Centers for Disease Control and Prevention, Atlanta, GA) yielded a minimum required sample size of 36 participants per group. To account for the biological variability in EMG signals and potential data attrition due to artifacts or non-compliance of the subjects, the sample size was rounded up to 40 participants per group, resulting in a total sample size of 80 participants (40 cricketers and 40 controls).

Selection criteria

Healthy university-level cricket players within the age range of 17-25 years, who played cricket for the last three years and practiced regularly daily for around three hours weekly, were considered eligible as cases. Subjects with no direct involvement in any sport were recruited as controls. Those having complaints of respiratory, cardiovascular, neurological, psychiatric, or systemic disorders or musculoskeletal disabilities, and those who refused to participate were excluded from the study.

Ethics consideration

Approval from the Institutional Ethics Committee (IEC) was obtained (IEC approval letter ref no. MGIMS/IEC/ANAT/90/2023, dated 25/03/2023). Written informed consent from all study participants was ensured before initiation of the study.

Study duration

The study was carried out within a duration of six months from 1 April 2023 to 1 October 2023 after due ethics approval on 25 March 2023.

Data sources and measurement of variables

Readings were documented in the data collection form (proforma), which included demographic and clinical data from all participants. The age of each participant was noted in years. Height measured bare feet in cms and weight in kgs on the Phoenix Height Weight Body Mass Index machine (Model: PBMI-200; Phoenix, New Delhi, India), which automatically displayed the body mass index (BMI).

Arm Anthropometrics 

Mid-upper arm circumference (MUAC): It is the circumference of the upper arm at the midpoint of the dominant upper arm.

Triceps skinfold (TSF): It was measured to the nearest 0.1 mm by SYGA Body Fat Analyzer Skinfold Calipers (Gujarat, India).

Derived anthropometric measures: The above measurements were used to derive the following indices:

Mid-upper arm area (MUAA): It is an estimation of the area of the upper arm derived as follows:

 $MUAA = (MUAC)^{2}/4\pi$

Mid-upper arm muscle circumference (MUAMC): It is an estimation of the circumference of the bone and muscle portions of the upper arm. It accounts for the thickness of subcutaneous fat that surrounds the muscle and is given as follows:

 $MUAMC = MUAC -(\pi \times TSF/10)$

Mid-upper arm muscle area (MUAMA): It is a measure of the area of the bone and muscle portions of the upper arm. It is derived as follows:

 $MUAMA = (MUAMC)^{2}/4\pi$

Corrected mid-upper arm muscle area (CMUAMA): It is an evaluation of the area of the muscle portions of the upper arm and is derived as follows:

 $CMUAMA = (MUAMC)^{2}-10/4\pi$

Mid-upper arm fat area (MUAFA): It is a measurement of the area of the fat portions of the upper arm and is given as follows:

$MUAFA = MUAA - MUAMA$

Arm fat index (AFI): It is derived as the fat percentage of the arm and is given as follows: 

$AFI = 100 \times MUAFA/MUAA$

Body fat percentage (BFP): For males, it is calculated as per the formula given by Lean et al. [16]:

$BFP = (1.52\times MUAC) +(0.336\times Age)-38.7$

Percentage lean body mass: $= 100 - BFP$

HGS and EMG

A galvanically isolated, high-performance biological amplifier, named BIO AMP (FE132; ADInstruments, Sydney, Australia), was optimized for measuring biological signals, such as sEMG, with high-quality noise specifications. It connects to the data acquisition PowerLab 8/35 system (ADInstruments, Sydney, Australia) at the core. sEMG was picked up by Ag-AgCl disposable electrodes applied on the surface lying just above the muscle of interest, and analysis was done by LabChart Pro software (ADInstruments, Sydney, Australia) from the raw data. When the skeletal muscle is made to contract, its myoelectric action is displayed as complicated wave patterns having an amplitude within a 0-10 mV range. LabChart’s root mean square (RMS) function was used to calculate the intensity of the EMG signal in real time.

The RMS values were expressed in millivolts (mV). A band-pass filter between 25 and 400 Hz was used to capture the raw EMG signal. RMS was sampled at a rate of 1,000 Hz, and it served as the best indicator of the physiological activity of the muscle motor units while the muscle is contracting [17].

A triangular smoothing window width of 0.5 seconds enabled RMS calculation from the raw EMG signal. The mean and maximum grip strength expressed in Newton (N) was measured for the dominant hand of the subjects by a grip force transducer with model no. MLT004/ST (ADInstruments, Sydney, Australia).

Various methodological steps were taken to minimize inter-individual variability and to reduce signal variability, including standardized electrode placement, identical recording equipment, uniform skin preparation, and consistent testing conditions with standardized posture and grip protocol across all participants. Additionally, the study involved age-matched participants within a narrow age range, which helped reduce physiological variability. It was ensured that every participant was investigated in the same duration of a day to maintain uniformity in the time-of-testing. Before coming to the laboratory, the subjects should have their last meal at least two hours prior and were advised to refrain from caffeinated drinks on the day of the investigation.

The subject was made to sit comfortably on a chair, and the dominant forearm of the subject was exposed. As per Table 1, the surface electrodes having a diameter of 1 cm were applied in alignment with the muscle as per the recommendations of sEMG for the noninvasive assessment of muscles (SENIAM) [18]. Red (positive) and white (negative) electrodes were placed over the proximal forearm on the belly of the muscle, while the ground electrode was on the anterior distal forearm. Silver or AgCl electrodes are preferred over others in order to minimize the effect of movements between electrodes and the skin surface on the potentials measured by electrodes at the skin surface. Because monopolar EMGs are unable to minimize crosstalk or interference caused by the neighboring power lines, from deeply situated motor units of the muscle studied or activity of distantly located muscles, bipolar electrodes are recommended over monopolar ones. A set of two chances with a gap of 20 seconds was given to each participant to record a maximum grip strength. The maximum HGS that corresponded to 100% of grip force and the best out of two chances was considered for calculation. A force of 30% of their maximum was used to grip the transducer in the dominant hand for about three to five minutes. To accustom the subject to the system for maintaining 30% of the grip, a practice trial was done with the non-dominant hand. LabChart's arithmetic function analysed the RMS of the EMG signal.

**Table 1:** Electrode placement and joint position during HGS testing cm: centimeters; HGS: handgrip strength

Parameter	Description
Inter-electrode distance	~2 cm (center-to-center)
Electrode orientation	Parallel to muscle fiber direction
Active electrodes placement	Over proximal one-third of the volar forearm, on the muscle belly, approximately 3–5 cms distal to the lateral epicondyle of humerus
Reference (ground) electrode	Anterior distal forearm over a relatively electrically neutral area
Skin preparation	Area cleaned with alcohol to reduce impedance
Shoulder position	Neutral position; shoulder adducted and internally rotated minimally (0° abduction, 0° flexion)
Elbow position	Flexed at approximately 90°
Forearm position	Supinated and resting comfortably on the arm support
Wrist position	Neutral (0-15° extension, 0° ulnar/radial deviation)
Hand position	Standard grip posture around the transducer
Trunk position	Upright sitting posture with back supported

Statistical analysis

An unpaired Student's t-test was employed to perform the statistical analysis for continuous variables. The mean and standard deviation of all studied continuous variables in normal distribution were estimated by using Statistical Product and Service Solutions (SPSS, version 21.0; IBM SPSS Statistics for Windows, Armonk, NY). Linear regression analyses were carried out using standard techniques. Pearson's correlation test was utilized for examining the correlation between variables. The correlation coefficient (r) was analysed for statistical significance. The level of significance was maintained at 95%.

## Results

The study included 40 cricketers in the age range of 17-25 years and 40 age-matched healthy subjects as controls. The mean age of cricketers was 20.08 ± 0.81 years, and those of controls were 20.00 ± 0.69 years. The descriptive statistics of the two study groups are listed in Table 2.

**Table 2:** Descriptive statistics of the study groups All data in the table are represented as mean ± SD. SD: standard deviation; cm: centimeters; kgs: kilograms; BMI: body mass index; kg/m^2^: kilograms per square meter; BP: blood pressure; mmHg: millimeters of mercury; NS: non-significant *All the p-values reported are based on Student's t-test between the case and control groups. p-value < 0.05 is considered to be statistically significant.

S. No.	Parameters	Cases (n=40), Mean ± SD	Controls (n=40), Mean ± SD	t statistic	*p-value
1	Age (Years)	20.08 ± 0.81	20.00 ± 0.69	0.3759	0.708 (NS)
2	Height (cms)	174.83 ± 5.81	172.72 ± 5.84	1.619	0.109 (NS)
3	Weight (kgs)	70.04 ± 12.28	65.88 ± 12.57	1.497	0.138 (NS)
4	BMI (kg/m^2^)	22.95 ± 4.21	22.06 ± 4.27	0.938	0.350 (NS)
6	Systolic BP (mmHg)	119.76 ± 7.81	120.66 ±8.45	0.513	0.609 (NS)
7	Diastolic BP (mmHg)	79.94 ± 8.44	77.78 ± 7.23	1.229	0.222 (NS)

It is evident from the table that the difference between means of age, height, weight, BMI, systolic BP, and diastolic BP was statistically non-significant (p > 0.05).

From Table 3, it is evident that the control group cases had significantly higher values of arm anthropometric parameters, such as MUAC, MUAA, MUAMC, MUAMA, CMUAMA, and BFP.

**Table 3:** Arm anthropometric parameters among the study groups All data in the table are represented as mean ± SD. SD: standard deviation; TSF: triceps skinfold; MUAC: mid-upper arm circumference; MUAMA: mid-upper arm muscle area; CUAMA: corrected mid-upper arm muscle area; MUAA: mid-upper arm area; MUAMC: mid-upper arm muscle circumference; MUAFA: mid-upper arm fat area; AFI: arm fat index; BFP: body fat percentage; cm: centimeters; cm^2^: square centimeters; mm: millimeters; S: significant; NS: non-significant; ES: extremely significant *All the p-values reported are based on Student's t-test between the controls and cases. p-value < 0.05 is considered to be statistically significant.

S. No	Parameters	Cases (n=40), Mean ± SD	Controls (n=40), Mean ± SD	t statistic	*p-value
1	TSF (mm)	17.75 ± 5.00	20.44 ± 5.12	2.377	0.019 (S)
2	MUAC (cm)	28.02 ± 2.03	26.16 ± 2.07	4.057	0.0001 (ES)
3	MUAA (cm^2^)	62.84 ± 9.22	54.83 ± 8.58	4.022	0.0001 (ES)
4	MUAMC (cm)	22.45 ± 2.63	19.74 ± 2.51	4.714	0.0001 (ES)
5	MUAMA (cm^2^)	40.67 ± 9.59	31.53 ± 7.72	4.695	0.0001 (ES)
6	CMUAMA (cm^2^)	39.87 ± 9.59	30.73 ± 7.72	4.695	0.0001 (ES)
7	MUAFA (cm^2^)	22.17 ± 5.92	23.30 ± 5.68	0.871	0.386 (NS)
8	AFI	35.69 ± 9.30	42.83 ± 9.40	3.415	0.001 (S)
9	Body Fat %	10.64 ± 3.19	7.78 ± 3.13	4.047	0.001 (S)

The HGS indices of males of both the study groups are depicted in the form of mean ± standard deviation, as given in Table 4.

**Table 4:** HGS indices of the study groups All data in the table are represented as mean ± SD. HGS: handgrip strength; S: significant; NS: non-significant; ES: extremely significant; RMS: root mean square; N: Newtons; mV: millivolts; SD: standard deviation *All the p-values were evaluated by Student's t-test between the study groups. p-value < 0.05 is considered to be statistically significant.

S. No	Parameters	Cases (n=40), Mean ± SD	Controls (n=40), Mean ± SD	t statistic	*p-value
1	Resting Heart Rate (Beats/min)	86.54 ± 12.11	95.88 ± 14.57	3.117	0.002 (S)
2	Max Grip Strength (N)	413.00 ± 78.81	334.52 ± 62.67	4.929	0.0001 (ES)
3	Mean Grip Strength (N)	109.70 ± 24.75	91.76 ± 17.23g	3.762	0.0003 (S)
4	Mean RMS (mV)	0.76 ± 0.22	0.64 ± 0.16	2.789	0.006 (S)

It is clearly evident from the above table that the differences in the HGS indices, namely, mean HGS and max HGS, when compared between the two groups, came out as statistically significant (Table 5). The difference between the resting heart rate of the two study groups was statistically significant (p = 0.002).

**Table 5:** Correlation analysis of the mean HGS between the case and control groups TSF: triceps skinfold; MUAC: mid-upper arm circumference; MUAA: mid-upper arm area; MUAMA: mid-upper arm muscle area; MUAFA: mid-upper arm fat area; AFI: arm fat index; cm: centimeters; cm^2^: square centimeters; mm: millimeters; S: significant; NS: non-significant *All the r values reported are based on Pearson's correlation test. p-value < 0.05 is considered to be statistically significant.

Variables	Cases (N=40)		Controls (N=40)	
	Correlation coefficient (r)	p-value	Correlation coefficient (r)	p-value
Weight	-0.04	0.8 (NS)	0.2	0.204 (NS)
TSF	-0.39	0.01 (S)	-0.09	0.570 (NS)
MUAC	0.10	0.52 (NS)	0.49	0.001 (S)
MUAA	0.11	0.48 (NS)	0.48	0.001 (S)
MUAMA	0.42	0.005 (S)	0.46	0.002 (S)
MUAFA	-0.36	0.01 (S)	0.10	0.52 (NS)
AFI	-0.40	0.008 (S)	-0.25	0.110 (NS)

On correlation analysis of the mean HGS as measured by a grip force transducer, it can be deduced that a statistically significant positive association with MUAMA (r = 0.42, p = 0.005) indicates an increase in the arm muscle area with a corresponding increase in the mean HGS value. A statistically significant negative correlation was found with TSF (r = -0.39, p = 0.01) and with AFI (r = -0.40, p = 0.008).

On correlation analysis of RMS calculated from sEMG (Table 6), it can be deduced that there was significant correlation with all arm anthropometrics, such as MUAC (r = 0.47), MUAA (r = 0.48), MUAMA (r = 0.41), mean HGS (r = 0.39), and max HGS (r = 0.47). The correlation of MUAFA with RMS was found to be non-significant.

**Table 6:** Correlation analysis of RMS EMG between the case and control groups N: Newtons; MUAC: mid-upper arm circumference; MUAMA: mid-upper arm muscle area; MUAA: mid-upper arm area; MUAFA: mid-upper arm fat area; cm: centimeters; cm^2^: square centimeters; S: significant; NS: non-significant *All the r values reported are based on Pearson's correlation test. p-value < 0.05 is considered to be statistically significant.

Variables	Cases (N=40)		Controls (N=40)	
	Correlation coefficient (r)	p-value	Correlation coefficient (r)	p-value
MUAC (cm)	0.47	0.001 (S)	0.4	0.008 (S)
MUAA	0.48	0.001 (S)	0.12	0.44 (NS)
MUAMA	0.41	0.007 (S)	0.05	0.753 (NS)
MUAFA	0.09	0.570 (NS)	-0.09	0.570 (NS)
Max. grip	0.47	0.001 (S)	0.57	0.0001 (ES)
Mean grip	0.39	0.01 (S)	0.40	0.008 (S)

## Discussion

Cricket is a physically challenging sport with multiple specializations that call for various abilities and levels of fitness. Cricket stands second only to soccer in popularity of sports being played worldwide [5]. The sport is becoming more and more popular each day, owing to its different match formats (one-day and five-day tests) and also due to the introduction of a new format, Twenty20 [19].

Hence, EMG evaluation of arm muscles in cricketers to ascertain the viability of sEMG methodology in clinical and research settings becomes all the more pertinent. Linking both elements (EMG and anthropometrics) with the hand grip function test of the dominant hand of a cricketer could provide a comprehensive, all-around fitness assessment at one go. The current research quantitatively evaluated arm anthropometrics, HGS, and RMS of surface EMG in male cricketers and age-matched controls.

The study results indicate that cricketers had greater means for height, MUAC, MUAA, MUAMA, BFP, and dominant HGS and lower means of TSF, MUAFA, and AFI than controls, depicting statistically significant differences of MUAC, MUAA, MUAMA, and dominant HGS between cricketers and the control group.

Although cricketers demonstrated significantly lower TSF and AFI, indicating reduced localized arm fat, the calculated BFP was higher compared to controls. This apparent discrepancy may be attributed to the predictive equation, which incorporates MUAC and may overestimate fat percentage in athletic individuals with greater muscle mass. Since MUAC reflects both muscle and fat components, increased muscle girth in cricketers could have contributed to higher estimated BFP, despite lower regional subcutaneous fat. Therefore, the elevated BFP should be interpreted cautiously in this athletic population.

The observation that BFP was statistically higher in cricketers compared to controls should not be interpreted as evidence that total body fat acted as a performance detractor in this cohort. The absolute values remained within a physiologically lean range for young males (mean: 10.64%).

With regards to MUAC, our values (28.02 ± 2.03 mm) agree with those reported in the literature by Koley et al. (20.91 ± 2.10 mm), Chilima et al. (25.00 ± 2.40 mm), and Adheke et al. (24.11 ± 2.58 mm) [10,11,14]. The MUAMA reported by Koley et al. was 35.19 ± 7.24 cm^2^ and by Chilima et al. was 25.00 ± 2.4 cm^2^, which is comparable to ours (i.e., 40.67 ± 9.59 cm^2^) [11,14].

The arm anthropometrics, such as MUAA and MUAFA, given by Koley et al., were 51.12 ± 12.62 and 15.94 ± 7.51, respectively, whereas our values were 40.67 ± 9.59 and 22.17 ± 5.92, respectively [11].

The differences in parameters could be attributed to the different regimes of training programs of cricketers, the effect of regular physical activity patterns, differences in arm muscle areas, locality (rural/urban), and, possibly, genetic predisposition.

Apart from investigating the dynamics of arm musculature, an earlier study has evaluated maximal voluntary isometric contraction as well [20]. Our results of HGS are in concordance with those of Adheke et al., Koley et al., and Chilima et al., who also concluded that better HGS, as compared to controls, helps the cricketers to produce effectively stronger force in the game [10,11,14].

The mean HGS of all subjects in our study was positively correlated with all arm anthropometric indices, which is also in agreement with all these studies. The correlation coefficient (r) of the mean HGS with MUAC, as determined by Chilima et al., was 0.45, with the arm muscle area of 0.39 and with TSF of 0.26 [14]. Similarly, in the study of cricketers by Koley et al., dominant HGS was significantly positively correlated with weight (r = 0.498), triceps skinfold (r = 0.278), arm muscle girth (r = 0.513), arm muscle area (r = 0.506), arm area (r = 0.493), and arm fat area (r = 0.326) [11]. These results are comparable to our positive correlation of the mean HGS with anthropometric variables such as MUAC (r = 0.10), MUAA (r = 0.11), and MUAMA (r = 0.42).

On the other hand, a negative correlation of HGS was obtained in our study with weight, TSF, and MUAFA, which is in contradiction to the positive correlation found by Koley et al. [11]. The correlation between MUAC and HGS obtained by Adheke et al., only in male athletes, was r = 0.54 (p < 0.001), which also supports our results [10]. The TSF in our study showed a statistically significant negative correlation with mean HGS (r = -0.39, p = 0.01), and this association may be partly due to reduced muscle mass owing to relatively greater fat mass. A statistically significant negative correlation was also found with AFI (r = -0.40, p = 0.008). Eventually, a decline in muscle mass will be associated with a decline in muscle strength.

Further, BFP did not demonstrate a negative correlation with either mean or maximum HGS. In contrast, arm-specific adiposity measures, such as TSF and AFI, showed inverse associations with grip strength. Therefore, our data suggest that localized arm fat distribution rather than total estimated body fat may have greater relevance to grip performance in this population.

Despite some research on arm muscle actions, there are no previous reports on EMG assessment with HGS in cricketers. After a thorough literature search, hardly any study has been found that performs an analysis of the function of the dominant hand in which the EMG signals and anthropometry are evaluated in conjunction.

Earlier studies have analysed the muscle electrical activity by placing electrodes over the skin through sEMG, which proved a more reliable technique to assess muscular force generation in comparison with dynamometry [5,13-15,20].

The RMS value obtained by sEMG in our subjects significantly correlated with all arm anthropometrics, such as MUAC (r = 0.47), MUAA (r = 0.48), MUAMA (r = 0.41), mean HGS (r = 0.39), and max HGS (r = 0.47), indicating a better electrical activity of the muscles in cricketers who have a greater muscle mass and hence a better grip strength as compared to controls. All these parameters were also found to be positively correlated with RMS EMG in the control group, as well with the exception of MUAFA (r = - 0.09). In this way, the key goal of the present study has been addressed by making the combined use of EMG and HGS along with arm anthropometrics, serving as a judicious way to evaluate the performance of the dominant hand of cricketers.

Limitations

This study has a few limitations to be acknowledged. The sample size was limited because of time and financial restraints of a short-term funded project. Additionally, the participants in the present study were not distinctively selected as bowlers or batters. As a methodological limitation, the EMG data were not normalized to maximum voluntary isometric contraction (MVIC) and were analyzed as raw RMS amplitudes in mV. Although standardized electrode placement and uniform testing conditions were maintained, the lack of normalization may limit inter-individual comparability due to potential variations in skin impedance and subcutaneous tissue thickness. The study design did not allow determination of whether increased RMS reflects enhanced neuromuscular efficiency, better recruitment of motor units, or merely greater force output.

Furthermore, this study tested HGS, EMG, and anthropometrics precisely of the dominant hand of just male cricketers. Further research investigating other muscles of the upper extremity of both sides, among both men and women, is warranted.

## Conclusions

Conclusively, the present study demonstrated that the mean and maximum HGS and RMS of EMG are significantly greater in cricket players in comparison to controls. These two variables also showed good correlation with the majority of the arm anthropometric variables recorded. It was a preliminary research proposing that a combination of sEMG, HGS, and arm anthropometrics could be a reliable tool, with optimal utility, for evaluating the activity of arm muscles in male cricketers. The values of mean HGS, max HGS, mean RMS EMG, and various arm anthropometrics derived from this research could serve as reference values for comparison of healthy and injured cricketers.
